# Beyond coupling coordination: configuration mechanisms of cultural–tourism–sports industry integration in regional development

**DOI:** 10.3389/fspor.2026.1792979

**Published:** 2026-05-07

**Authors:** Biaofeng Zhang, Mingyang Yu, Yu Lu, Jiahui Li

**Affiliations:** 1 Department of Physical Education, Kunsan National University, Gunsan, Republic of Korea; 2 School of Humanities, Shangqiu University, Shangqiu, China

**Keywords:** configuration mechanism, coupling coordination, culture tourism sports industry, fuzzy set qualitative comparative analysis (fsQCA), multi industry integration, the middle and lower reaches of the Yellow River region

## Abstract

**Introduction:**

Against the backdrop of economic restructuring and changing consumption patterns, the integrated development of the cultural, tourism, and sports industries has become an important driver of regional growth. Existing studies mainly focus on measuring the degree of coordination among these industries, while paying limited attention to the mechanisms through which coordination is achieved. This study aims to identify the dominant patterns of coordinated development among the cultural, tourism, and sports industries and to explore the underlying mechanisms that shape such coordination.

**Methods:**

Using provinces in the middle and lower reaches of the Yellow River as an empirical case, this study assesses the development levels and coordination status of the cultural, tourism, and sports industries to identify key constraints on their joint development. A configurational comparative approach is employed to examine multiple pathways shaped by differences in economic conditions, industrial structure, and innovation capacity across provinces.

**Results:**

The results show that the cultural, tourism, and sports industries are closely interconnected in promoting regional development, but their level of synergy evolves gradually and varies significantly across provinces. No single factor determines the level of coordinated development. Instead, similar outcomes can be achieved through different combinations of economic, structural, and innovation-related conditions.

**Discussion:**

The findings indicate that the coordinated development of the cultural, tourism, and sports industries follows multiple pathways, highlighting the importance of differentiated development strategies tailored to regional characteristics. The study contributes to a better understanding of the mechanisms underlying industrial integration and provides practical insights for promoting high-quality regional development.

## 1 Introduction

In the context of the transition toward a service-oriented economy, the continued growth of experience-based consumption, and sustainability-oriented policies, the integration of the cultural, tourism, and sports industries has emerged as an important strategic pathway for regional development (1, 2). Globally, the cultural, tourism, and sports industries have become key drivers of employment creation, regional branding, and social well-being, while also reshaping development trajectories and economic structures (3, 4). As traditional development models face increasing constraints, multi-industry integration has gained importance as a means of enhancing regional resilience and achieving high-quality development (5).

From a theoretical perspective, multi-industry integration is a dynamic evolutionary process in which previously distinct industries become increasingly interconnected through the combined influence of technological convergence, diversified demand, and policy guidance (6). Research in evolutionary economic geography and regional innovation systems suggests that stronger inter-industry linkages facilitate knowledge spillovers, resource sharing, and collective learning, thereby improving regional competitiveness and adaptive capacity (7–9). In this sense, multi-industry integration goes beyond simple sectoral collaboration and constitutes a complex system in which different industries co-evolve and jointly shape regional development outcomes (10).

Within this framework, the cultural, tourism, and sports industries form a highly complementary structural combination. They are closely linked through shared consumption spaces, symbolic value creation, and experiential production processes, giving them a natural advantage in achieving synergistic development (11, 12). At the same time, sports-related activities—such as events, venues, and leisure services—have been shown to stimulate tourism demand and support urban regeneration, generating significant spillover effects for related industries (13, 14). Recent studies further indicate that cross-sector integration among the cultural, tourism, and sports industries supports economic diversification and enhances regional resilience, particularly in regions undergoing structural transformation (15, 16).

Despite growing evidence of the benefits of such integration, existing research remains limited in several respects. First, most studies focus on bilateral relationships—such as culture–tourism or sports–tourism—while paying insufficient attention to the overall interaction among all three industries as a system (17, 18). Second, prior research relies mainly on descriptive approaches or single-equation models, which are poorly suited to capturing the nonlinear and context-dependent nature of multi-industry integration processes (19). Because regional development outcomes result from the joint influence of multiple factors, linear analytical methods may obscure the diverse pathways through which similar levels of industrial synergy are achieved (20).

Current policy in China explicitly identifies the integration of the cultural, tourism, and sports industries as a key approach to promoting high-quality regional development. Domestic studies have measured and compared regional development levels and spatial patterns by constructing composite indicator systems and applying coupling coordination models (21–23). However, coupling coordination is often treated merely as an outcome indicator, with limited attention given to the mechanisms through which coordination is achieved (24). As a result, systematic theoretical explanations and empirical evidence on the underlying mechanisms and configuration pathways of multi-industry integration remain insufficient.

Recent methodological advances suggest that configurational approaches offer distinct advantages in addressing these limitations. For example, fuzzy-set qualitative comparative analysis (fsQCA) can identify multiple functionally equivalent pathways, revealing how different factors combine or substitute for one another (25, 26). By applying this method to the study of regional industrial integration, this research moves beyond single-factor explanations and provides a more comprehensive account of the complexity and heterogeneity of cultural–tourism–sports integration processes (27).

Accordingly, this study develops a configurational analytical framework to systematically examine the coordinated development of the cultural, tourism, and sports industries across provinces in the middle and lower reaches of the Yellow River. By combining coupling coordination analysis with fsQCA, the study not only identifies the overall pattern of industrial synergy across provinces but also explores the multiple pathways through which high-level integration is achieved under different regional conditions. The key contribution of this study lies in shifting from static measurement to mechanism-oriented explanation, thereby enriching the theoretical understanding of multi-industry integration and providing empirically grounded insights for regional policy design.

Based on this approach, the study addresses the following research questions:

**RQ1:** How does the coupling coordination level among the cultural, tourism, and sports industries evolve across provinces and stages in the middle and lower reaches of the Yellow River?
**RQ2:** What key factors constrain the coordinated development of the multi-industry system?
**RQ3:** What different configurational pathways lead to high levels of coupling coordination under heterogeneous regional conditions?

## 2 Theoretical foundations

Regional multi-industry integration is not a linear process, nor is it driven by a single factor. Instead, it emerges from the interaction of multiple industrial elements under specific spatial, institutional, and developmental conditions. To systematically explain the formation logic of coordinated development among the cultural, tourism, and sports industries in the middle and lower reaches of the Yellow River, this study draws on cluster theory, system coupling theory, and sustainable development theory as its theoretical foundation.

### 2.1 Cluster theory: spatial and organizational foundations of multi-industry integration

Cluster theory suggests that industries with technological linkages or complementary demand structures tend to develop synergistic advantages through shared resources, knowledge spillovers, and networked specialization when located in close proximity. This perspective provides a key basis for understanding multi-industry integration. Research in evolutionary economic geography shows that coordinated development driven by multiple factors, rather than isolated industry growth, is the main mechanism behind industrial co-evolution and structural upgrading at the regional level (28).

The concept of industrial clusters has expanded from homogeneous concentration toward coordinated development across multiple industries (29). Across consumption spaces, content creation, and experience-based value generation, the cultural, tourism, and sports industries are highly interconnected. Their integration is largely shaped by platform-based organizational forms, factor mobility, and regional institutional coordination (30). Accordingly, this study adopts industrial clustering as an analytical entry point and explains the differentiated pathways of coordinated cultural–tourism–sports development in the middle and lower Yellow River region from both spatial and organizational perspectives.

### 2.2 System coupling theory: the dynamic logic of multi-industry co-evolution

System coupling theory holds that continuous interaction among subsystems leads to coordinated evolutionary structures, with outcomes determined by the degree of alignment among subsystems and the efficiency of resource allocation. At the regional level, this theory has been widely applied to explain the transition from simple industrial co-location to functional coordination, where industries evolve from basic coexistence toward differentiated yet complementary roles (31).

The cultural, tourism, and sports industries are closely linked in terms of development objectives, market demand, and resource dependence, resulting in relatively high levels of coupling. However, strong coupling does not automatically translate into high-quality coordination, which is also shaped by factors such as industrial structure, innovation capacity, and the institutional environment (32). This highlights the nonlinear and context-dependent nature of multi-industry collaboration, making single-factor or linear analytical approaches insufficient for capturing its underlying mechanisms. Building on this insight, the study focuses on the cultural–tourism–sports system in the middle and lower Yellow River region and employs a configurational approach to explain how coordinated outcomes emerge through multiple causal pathways across different regional contexts (33).

### 2.3 Sustainable development theory: a normative and long-term perspective

Sustainable development theory emphasizes the internal balance among economic growth, social welfare, and structural optimization, offering a normative framework for analyzing multi-industry integration. Existing studies show that industrial coordination and resource reallocation can enhance regional economic resilience and reduce dependence on single development paths (34).

The cultural, tourism, and sports industries combine economic and public-good attributes. Their integration expands consumption opportunities, strengthens regional attractiveness, and supports local identity formation and public service provision. Especially during periods of structural transition, multi-industry coordination serves as a key mechanism for achieving high-quality and sustainable regional development (35). From a sustainability perspective, examining cultural–tourism–sports integration helps avoid an overemphasis on short-term growth or single-factor explanations, while placing greater emphasis on the long-term stability and structural soundness of different collaboration models.

Taken together, cluster theory highlights the spatial and organizational foundations of integration, system coupling theory explains the dynamic coordination process, and sustainable development theory provides long-term value criteria for different integration pathways among the cultural, tourism, and sports industries. Together, these theories converge on a central insight: coordinated development among the cultural, tourism, and sports industries does not follow a single optimal model but emerges from different configurations of factors shaped by regional institutional contexts. Accordingly, this study conceptualizes cultural–tourism–sports coordination as a configurational outcome and applies fuzzy-set qualitative comparative analysis (fsQCA) to identify multiple causal pathways leading to high levels of coupling coordination. Within this framework, the study advances coupling coordination research from descriptive assessment toward mechanism-oriented explanation, offering new theoretical perspectives and methodological support for research on multi-industry integration.

## 3 Methods

### 3.1 Construction of the indicator system

#### 3.1.1 Principles for indicator selection

To better capture the overall development levels of the cultural, tourism, and sports industries across provinces in the middle and lower reaches of the Yellow River, as well as their coordination characteristics, indicator selection followed two core principles: theoretical relevance and data availability. According to input–output theory, industrial development reflects the joint effects of factor inputs and economic and social outputs. This framework has been widely applied in studies of multi-industry coordination and provides a solid analytical basis for measuring inter-industry relationships.

Accordingly, this study conducted a systematic review of literature published between 2010 and 2019 on industrial development, integration performance, and cultural–tourism–sports collaboration, with particular attention to the evaluation indicators used in key studies, in order to ensure methodological rigor. Based on this review, indicators with consistent statistical definitions and continuous data availability were selected to ensure comparability across regions and time periods.

#### 3.1.2 Structure and content of the indicator system

In constructing the indicator system, this study considered both industrial categories and stages of development. For each of the cultural, tourism, and sports industries, separate input and output subsystems were established, forming an “industry–input–output” analytical framework. This framework allows not only for the assessment of basic development conditions and performance outcomes, but also for an examination of interactions within the multi-industry system.

Overall, the coupling coordination evaluation system for the cultural, tourism, and sports industries in the middle and lower Yellow River region consists of six first-level indicators and twenty-one second-level indicators. Third-level indicators capture infrastructure provision, financial support, market size, employment capacity, and economic performance. Indicator weights were then determined using the entropy method (see Table 1). The resulting indicator system effectively reflects both the development levels and the co-evolutionary characteristics of the three industries across regions, providing reliable variables for subsequent coupling coordination measurement and pathway analysis.

**Table 1:** Evaluation indicator system and weights for coupling coordination among the cultural, tourism, and sports industries in the middle and lower reaches of the Yellow River.

Primary indicator	Secondary indicator	Attribute	Weight
Cultural Industry Input	Number of Libraries(C1)	+	0.049
	Number of Cultural Centers(C2)	+	0.055
	Proportion of Cultural Expenditure in Fiscal Budget(C3)	+	0.048
Cultural Industry Output	Number of Cultural Market Operators(C4)	+	0.048
	Number of Cultural Industry Employees(C5)	+	0.048
	Operating Profit of Cultural Industry(C6)	+	0.046
Tourism Industry Input	Number of Travel Agencies(C7)	+	0.046
	Number of Star-rated Hotels(C8)	+	0.048
	Number of A-level Tourist Attractions(C9)	+	0.052
Tourism Industry Output	Number of Tourism Employees(C10)	+	0.052
	Number of Domestic Tourists(C11)	+	0.050
	Number of International Tourists(C12)	+	0.047
	Domestic Tourism Revenue(C13)	+	0.050
	International Tourism Earnings(C14)	+	0.048
Sports Industry Input	Sports Fiscal Expenditure(C15)	+	0.044
	Number of Sports Social Organizations(C16)	+	0.045
	Number of Classified Athletes(C17)	+	0.051
	Number of Classified Referees(C18)	+	0.043
Sports Industry Output	Number of Sports System Employees(C19)	+	0.044
	Operating Income of Sports Institutions(C20)	+	0.045
	Sports Lottery Sales(C21)	+	0.043

The entropy method is an objective weighting approach based on information entropy theory. It captures variation among indicators and reduces subjectivity in weight assignment (36). Under this method, indicator weights are determined by the degree of variation: indicators with greater variability contain more information and are assigned higher weights in the composite evaluation. Accordingly, this study applies the entropy method to weight the indicators, following the steps outlined below.

The formula for indicator standardization is as follows:

Prior to the entropy weight calculation, all original indicator data were standardized to eliminate dimensional disparities across variables. As all indicators in this study are benefit-oriented (i.e., higher values correspond to higher levels of development), the min–max normalization method was employed to process the data. Accordingly, the calculation procedures for indicator standardization, entropy weighting, subsystem composite development level, and obstacle degree are presented in Equations (1–7) and Equations (11–13).

$$\mathrm{Positive}\;\mathrm{indicator}:\;x_{ij}^{{}^{\prime }}=\frac{x_{ij-min(x_{j})}}{max(x_{j})-min(x_{j})}$$ (1)

$$\mathrm{Negative}\;\mathrm{indicator}:\;x_{ij}^{{}^{\prime }}=\frac{\max(x_{j})-x_{ij}}{max(x_{j})-min(x_{j})}$$ (2)

$$p_{ij}=\frac{x_{ij}^{{}^{\prime }}}{\sum_{i=1}^{n}x_{ij}^{{}^{\prime }}}$$ (3)

$$e_{j}=-k\overset{n}{\underset{i=1}{\sum }}p_{ij}\ln(\;p_{ij})$$ (4)

$$d_{j}=1-e_{j}$$ (5)

$$w_{j}=\frac{d_{j}}{\sum d_{j}}$$ (6)

Here, $p_{ij}$ denotes the proportion of the standardized value of indicator *j* in region *i*; $e_{j}$ represents the information entropy of indicator *j*; $d_{j}$ is the information utility (or divergence coefficient); and $w_{j}$ denotes the weight assigned to indicator *j*.

### 3.2 Coupling coordination model

Based on the determined indicator weights, composite development scores for each subsystem are calculated using the following formula:

$$u_{i}=\overset{m}{\underset{\;j=1}{\sum }}w_{j}x_{ij}^{{}^{\prime }}$$ (7)

In this equation, $u_{i}$ represents the development level of the system in region *i*; $w_{j}$ is the weight of indicator *j*; and $x_{ij}^{\prime }$ denotes the standardized value of indicator *j* in region *i*.

### 3.3 Coupling coordination degree model

To assess interactions among the cultural, tourism, and sports industry systems and their level of coordinated development, this study applies the coupling coordination degree model. The coupling degree measures the strength of interdependence among subsystems and is calculated as follows:

$$C={\left[\frac{u_{1}u_{2}u_{3}}{{\left(\frac{u_{1+}u_{2+}u_{3}}{3}\right)}^{3}}\right]}^{\frac{1}{3}}$$ (8)

Here, *C* denotes the coupling degree, reflecting the intensity of interaction between three systems, while $U_{1},\;U_{2}$ and $U_{3}$ represent the composite development levels of the three subsystems, respectively.

On this basis, a comprehensive coordination index is constructed using the following formula:

$$T=\alpha U_{1}+\beta U_{2}+\gamma U_{3}$$ (9)

In this study, the weighting coefficients α, β, and γ are assumed to be equal, reflecting the equal importance of the three subsystems—culture, tourism, and sports—in the coordinated development process. Accordingly, the weights are set as α = β = γ = 1/3

This study conceptualizes culture, tourism, and sports as three mutually embedded subsystems within the broader framework of regional integrated development. Drawing on system coupling theory, when no subsystem can be theoretically or empirically demonstrated to exercise hierarchical dominance, the adoption of a symmetry assumption is methodologically justified. Moreover, national policy frameworks in China position culture, tourism, and sports as three parallel pillars of regional development, emphasizing balanced and coordinated advancement rather than the primacy of any single sector. In light of this context, equal weighting is employed to avoid introducing subjective bias into the measurement process.

To assess the robustness of the weighting assumption, sensitivity analyses were conducted under alternative weighting schemes (0.4–0.3–0.3; 0.3–0.4–0.3; 0.3–0.3–0.4). Based on the recalculated coupling coordination degrees, provincial rankings remained highly consistent across different weighting scenarios. Compared with the baseline model, Spearman rank correlation coefficients all exceeded 0.90. In addition, the classification of provinces by coordination level showed no variation under the alternative specifications. These findings suggest that the study's conclusions are robust to moderate adjustments in subsystem weights.

The coupling coordination degree is then obtained by combining the coupling degree with the comprehensive coordination index, as shown in the following equation:

$$D=\sqrt{C*T}$$ (10)

The value of *D* ranges from 0 to 1, with higher values indicating a higher level of coordinated development among the cultural, tourism, and sports industries. Based on the calculated results, the coupling coordination degree is further classified into distinct levels to characterize different stages of coordinated cultural–tourism–sports development across regions (see Table 2).

**Table 2:** Classification of coupling coordination levels.

Coordination range	Coordination type	Description
0.0–0.3	Low Coordination	Significant imbalance in development between systems.
0.3–0.5	Moderate Coordination	Preliminary linkage between the three systems.
0.5–0.7	Good Coordination	Synergistic effects are evident.
0.7–1.0	High Coordination	Systems are highly integrated in their development.

### 3.4 Obstacle degree model

To identify key factors constraining coordinated development within the system, this study applies the obstacle degree model. This model evaluates the extent to which each factor hinders system coordination by jointly considering indicator weights and deviation levels, as expressed in the following equation:

$$I_{j}=w_{j}*x_{ij}^{{}^{\prime }}$$ (11)

$$F_{j}=1-x_{ij}^{{}^{\prime }}$$ (12)

$$O_{j}=\frac{I_{j}*F_{j}}{\sum (I_{j}*F_{j})}*100\text{\%}$$ (13)

In this formulation, $I_{j}$ denotes the contribution of indicator $j\;F_{j}$ represents its deviation degree; and $O_{j}$ indicates the obstacle degree of indicator *j*. A higher value of $O_{j}$ indicates a stronger constraining effect of the corresponding indicator on coordinated system development.

The calculation of obstacle degree was conducted at the secondary indicator level. The obstacle degree of each indicator is derived from the weight obtained through the entropy method and the standardized numerical value, representing the degree of deviation and relative contribution to the optimal development state. The reference for obstacle diagnosis is based on the overall coupling coordination degree of the comprehensive system of culture, tourism, and sports. Specifically, the analysis aims to identify the influencing factors that constrain the coordinated development at the system level, rather than evaluating the obstacle factors within a single industry.

### 3.5 Data sources and processing

Given differences in statistical definitions across years and regions in the sports sector, as well as the substantial disruption caused by the COVID-19 pandemic during 2020–2023, this study focuses on the period 2010–2019 to ensure data completeness and comparability. Data were primarily obtained from the China Statistical Yearbook, China Tourism Statistical Yearbook, China Sports Statistical Yearbook, as well as provincial statistical yearbooks and official statistical bulletins. The empirical analysis covers five provinces in the middle and lower reaches of the Yellow River—Inner Mongolia, Shaanxi, Shanxi, Henan, and Shandong—with annual data systematically collected for the cultural, tourism, and sports industries in each province. After stage-based averaging, the resulting values were aggregated at the provincial level. Therefore, the fsQCA analysis treats each province as a single case, resulting in a dataset of five cases corresponding to the five provinces in the study region.

Although this study is based on a small sample, there are significant differences among different provinces, specifically reflected in aspects such as economic development level, industrial structure, innovation capability, and fiscal strength. Shandong Province is characterized by diversified industrial structure and relative development; Henan Province is a province with rapid development of the tertiary industry and a huge market demand; Shaanxi Province is characterized by innovation-driven economic growth and profound historical and cultural heritage; Inner Mongolia is characterized by frontier development mode and relatively weak innovation capability; Shanxi Province is characterized by typical resource-dependent industrial transformation. These structural differences provide a substantial cross-case variation basis for configuration comparison in the regional context.

During data preprocessing, missing values in non-structural indicators were supplemented using interpolation methods. A small number of missing observations (11 in total, approximately 1.05% of the dataset) were supplemented using linear interpolation based on adjacent years. The specific indicators, provinces, and years for which interpolation was applied are reported in Appendix Table A1.

Moreover, given the persistence and time-lag effects of industrial structure and integration, policy impacts are unlikely to materialize immediately. The analysis therefore divides the study period into five stages: 2010–2011, 2012–2013, 2014–2015, 2016–2017, and 2018–2019. Stage-based analysis ensures temporal consistency and comparability, thereby providing more robust empirical support for the study's conclusions.

Linear interpolation is generally appropriate when isolated observations are missing for certain indicators in specific years, provided that data for the adjacent years are available. In this study, interpolated values account for less than 5% of the total observations. To assess the potential impact of interpolation on the overall findings, robustness checks were conducted using non-interpolated data. The results indicate no substantive differences in temporal evolution trends, provincial rankings, or the classification of coordination levels. As the fsQCA analysis is based on stage-averaged values rather than single-year observations, the influence of interpolation on the configurational results can be considered negligible.

## 4 Results

**Figure 1:**
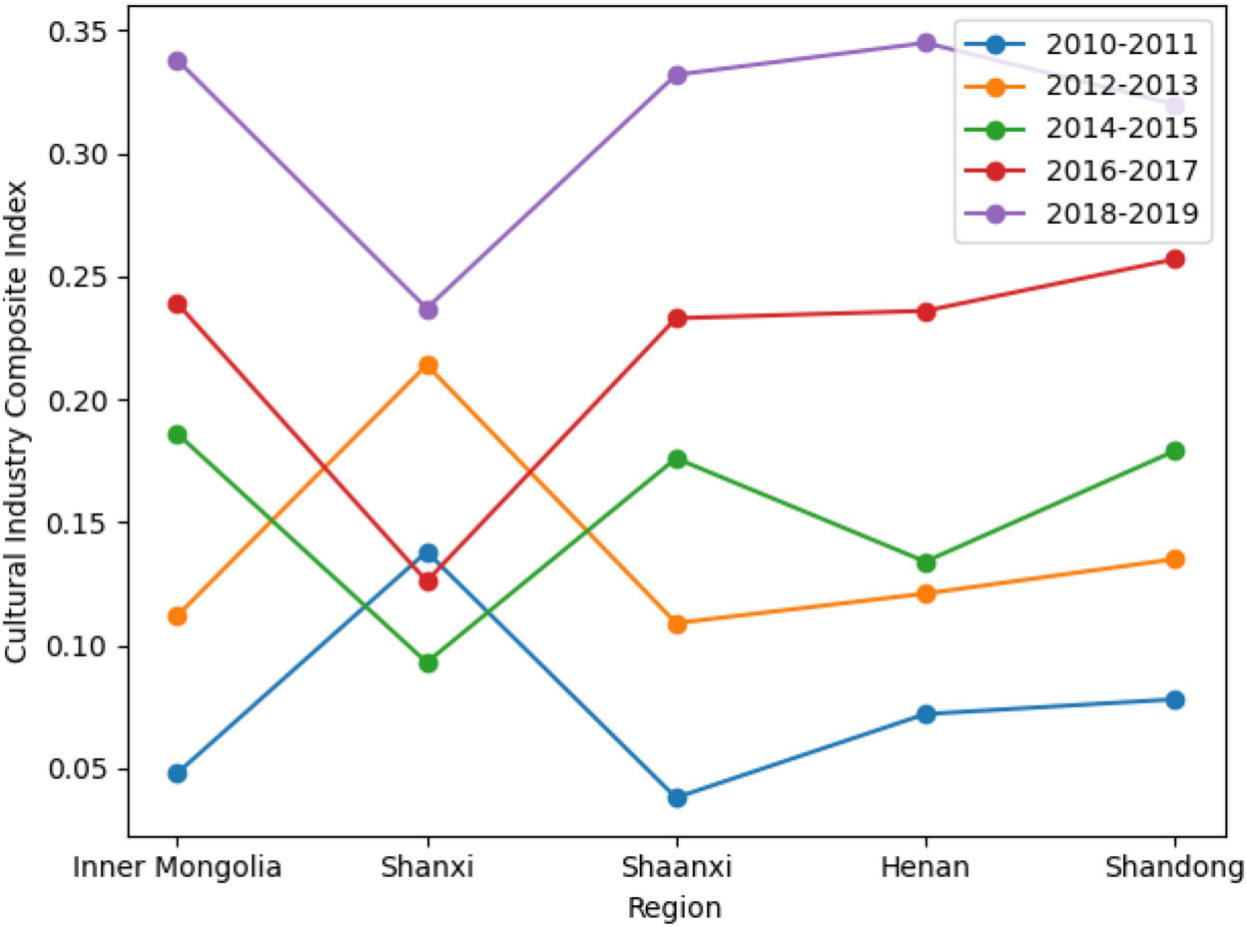
Temporal evolution of the composite index of the cultural industry across regions.

**Figure 2:**
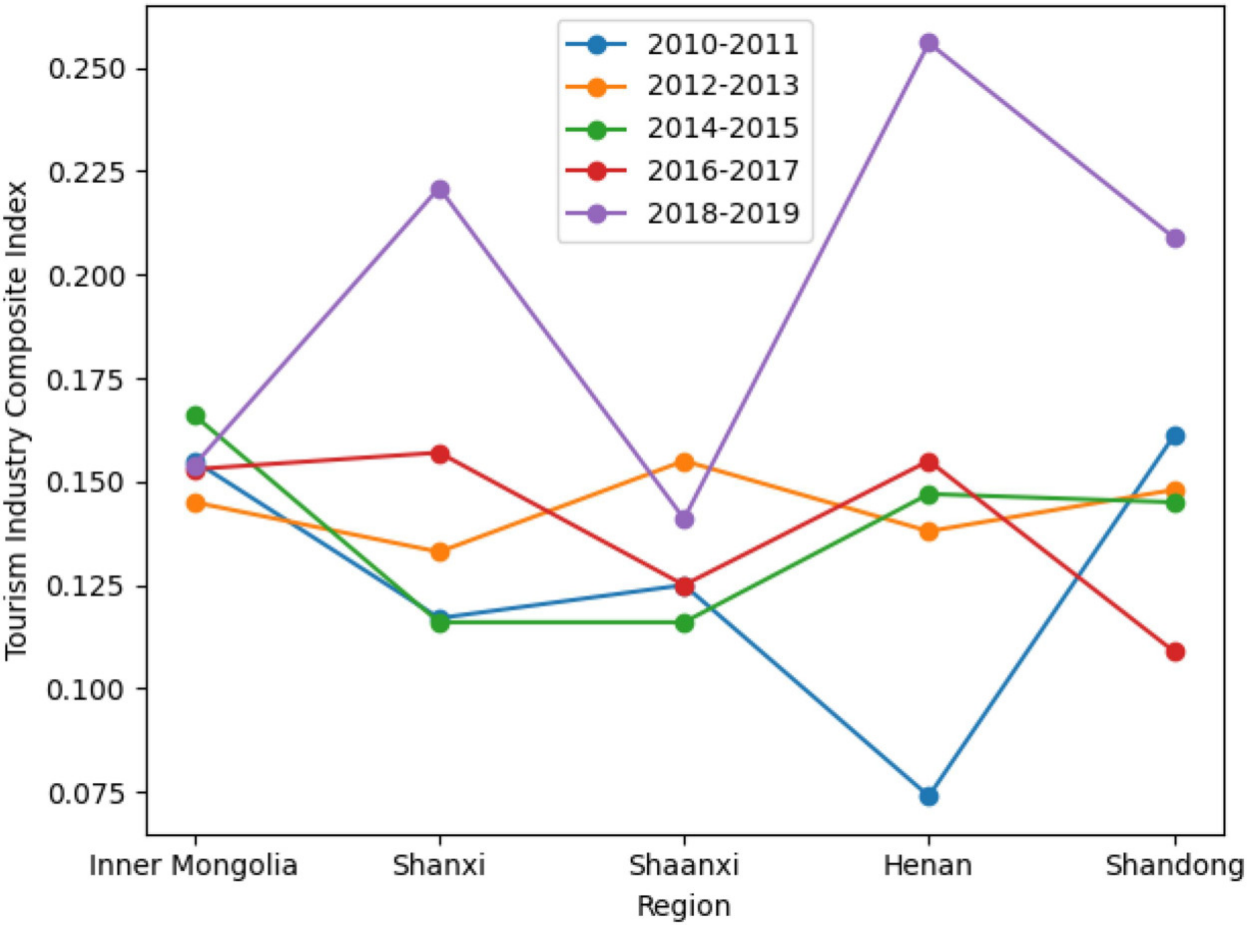
Stage-based evolution of the tourism industry composite index across regions.

**Figure 3:**
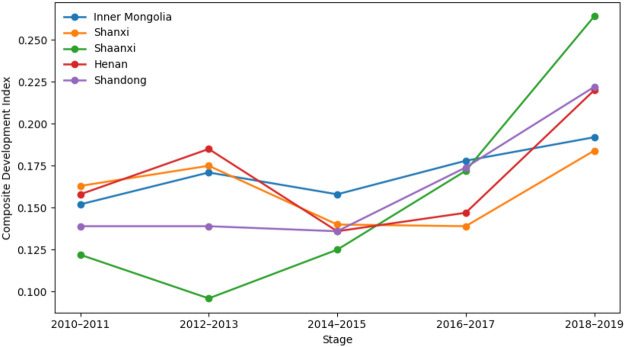
Stage-based evolution of the sports industry composite index across regions.

Based on the composite development indices of the cultural, tourism, and sports industries, this section analyzes their spatial evolution across the five provinces from 2010 to 2019 (Figures 1–3). By comparing development trajectories across stages and regions, the analysis identifies stage-specific characteristics, spatial differentiation patterns, and inter-industry heterogeneity. These findings provide a solid empirical foundation for subsequent coupling coordination analysis and the exploration of multiple development pathways.

### 4.1 Spatiotemporal evolution of the composite development index

#### 4.1.1 Overall temporal patterns

From a temporal perspective, the composite development indices of the cultural, tourism, and sports industries show an overall upward trend between 2010 and 2019, though provinces differ markedly in growth trajectories and volatility. The cultural industry exhibits steady and cumulative growth, with relatively smooth development paths across provinces. Tourism shows a combination of stable growth and pronounced fluctuations, indicating high sensitivity to external conditions. In contrast, the sports industry displays strong stage-specific dynamics, with slow early growth followed by accelerated expansion in later periods.

Overall, the three industries differ substantially in development stages, structural characteristics, and responses to external shocks across the five provinces.

#### 4.1.2 Regional differences and spatial patterns

Spatially, all three industries have shifted from relative balance toward increasing regional differentiation, though the degree of divergence varies by industry. Regional disparities are modest in the cultural industry, with steady growth across provinces, while tourism exhibits pronounced spatial differences. The sports industry shows clear stratification in later periods, with Shaanxi and Henan experiencing substantially higher growth than other provinces. Inconsistent regional rankings across industries indicate that regional development is not driven by a single leading sector.

#### 4.1.3 Comparative insights

Comparatively, the cultural industry serves as a stable foundation, tourism is characterized by cyclical fluctuations and external dependence, and the sports industry is strongly influenced by policy support and integration dynamics. The asynchronous evolution of the three industries across time and space suggests that integration is not a linear aggregation process but the outcome of interaction through differentiated pathways under specific conditions.

### 4.2 Evolution of coupling degree and coupling coordination

**Figure 4:**
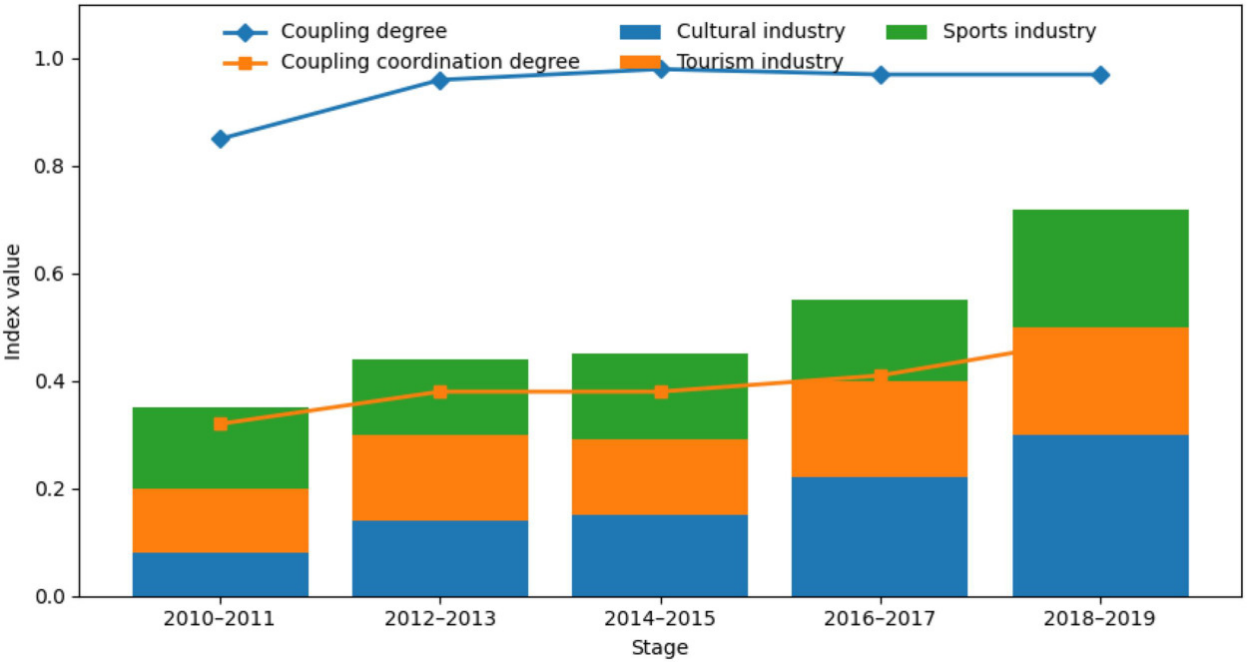
Evolutionary characteristics of coupling degree and coupling coordination among the culture–tourism–sports industries.

Building on the composite development index analysis, this section further examines the evolution of coupling degree and coupling coordination among the cultural, tourism, and sports systems. By tracking changes in overall development levels and inter-industry interactions, the analysis systematically depicts the evolution of industrial linkages and coordinated development (Figure 4).

#### 4.2.1 Temporal evolution of overall development and coupling relationships

Figure 4 illustrates the temporal evolution of the composite development indices of the cultural, tourism, and sports industries, together with changes in coupling degree and coupling coordination during the study period. Overall, during the periods 2010–2011 and 2018–2019, the overall development level of the three industries in the middle and lower reaches of the Yellow River increased steadily, indicating a gradual consolidation of the regional industrial foundation.

From an internal perspective, the cultural industry maintained stable and continuous growth throughout the study period and gradually became a key contributor to the increase in the composite index in later stages. Both the tourism and sports industries also exhibited upward trends, although growth rates were relatively moderate and accompanied by varying degrees of fluctuation across stages. These patterns indicate that improvements in overall development were not driven by a single leading industry, but rather resulted from the joint contribution and coordinated advancement of all three industries.

#### 4.2.2 Evolution of the coupling degree

The coupling degree among the cultural, tourism, and sports industries remained consistently high throughout the study period, with only minor fluctuations in certain stages. This indicates the presence of stable and close structural linkages among the three industries. The sustained high coupling suggests strong interdependence in terms of resource allocation, market demand, and functional specialization, reflecting the emergence of an integrated multi-industry development system.

#### 4.2.3 Evolution of the coupling coordination degree

Over the study period, the coupling coordination degree exhibited a steady upward trend. Although coordination was relatively low in 2010–2011, it increased continuously and steadily thereafter. A combined assessment of coupling degree and coordination trajectories suggests that strong structural linkages among the three industries were established early, while the quality and efficiency of coordinated development lagged behind. As a result, improvements in coordination became evident only in later stages.

The continuous rise in coupling coordination reflects a transition from a “high coupling–low coordination” pattern to a “high coupling–high coordination” pattern in the region. This transition highlights the dynamic nature of multi-industry interaction and shows that strong linkages alone are insufficient to achieve high-level coordination. Sustained improvement in overall development levels is the key factor driving higher coupling coordination.

Overall, the cultural–tourism–sports system in the middle and lower reaches of the Yellow River is characterized by steadily rising development levels, persistently high coupling strength, and continuously improving coordination. This suggests that high coupling coordination emerges from cumulative multi-industry development combined with improvements within individual industries, rather than from strong inter-industry linkages alone.

#### 4.2.4 Coupling coordination Status

**Figure 5:**
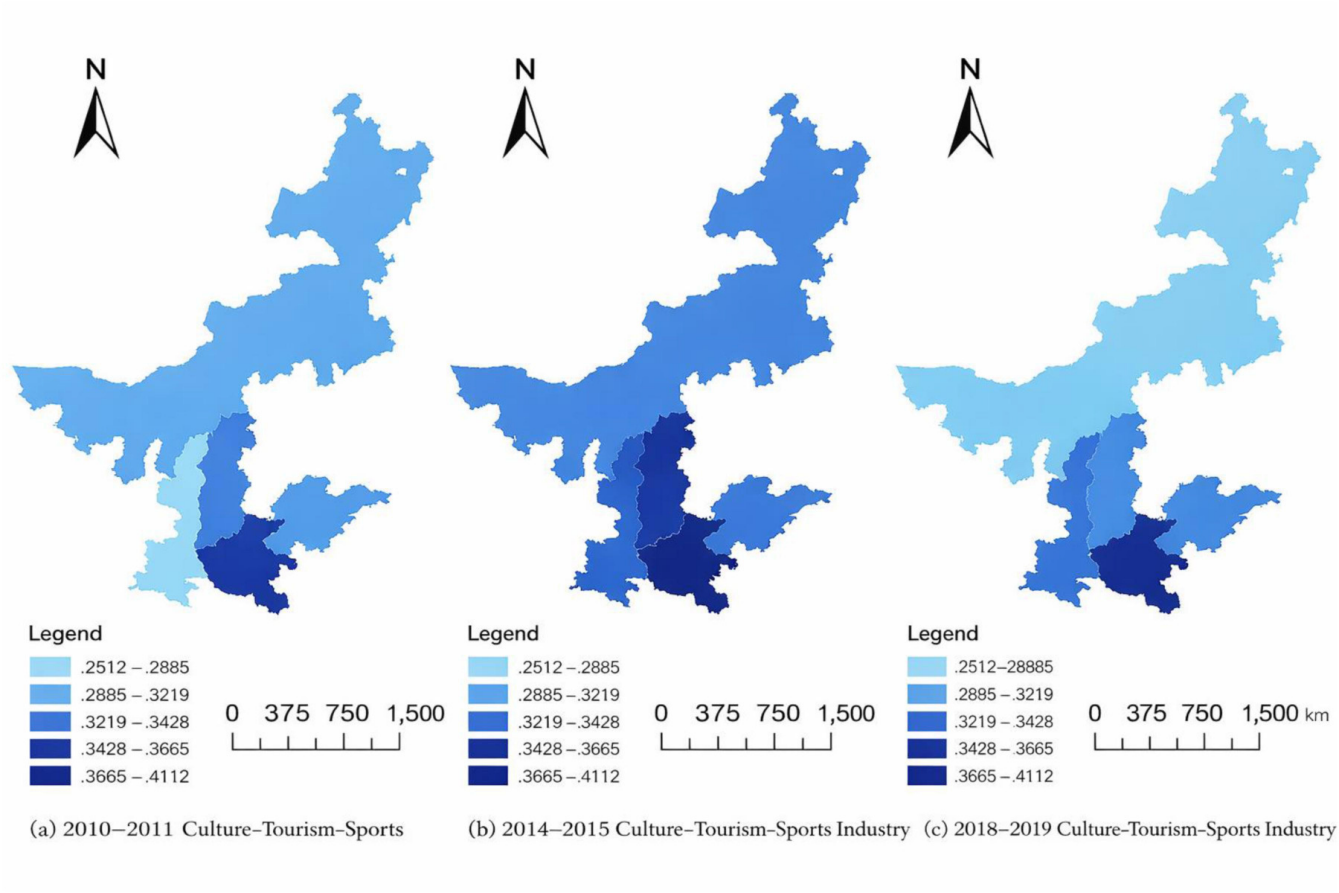
Spatial distribution of the culture-tourism-sports integration index across different periods. **(A)** 2010–2011 period; **(B)** 2014–2015 period; **(C)** 2018–2019 period.

Based on the composite development indices, a coupling coordination model is applied to assess the level of coordinated development among the three industries across provinces in the middle and lower reaches of the Yellow River. This approach enables a systematic evaluation of regional coupling coordination levels. Figure 5 depicts the spatial and temporal evolution of coupling coordination, with color gradients indicating differences in coordination levels across the region. Darker colors represent higher levels of coupling coordination. These results are derived using Equations (8)–(10) defined in the coupling coordination model.

Overall, during the study period, the coupling and coordination among the cultural, tourism, and sports industries in the middle and lower reaches of the Yellow River exhibited a clear evolutionary trend, progressing from lower to higher levels. In the 2010–2011 period, coordination in most provinces remained at low to moderate levels, indicating that although basic structural linkages had formed among the three industries, coordination efficiency and quality were still limited. As development advanced, coordination levels across provinces improved markedly after 2014–2015, reflecting a transition from a “high coupling–low coordination” pattern to a “high coupling–high coordination” pattern.

Spatially, coupling coordination displayed pronounced regional disparities, while also showing signs of gradual convergence. Provinces with stronger economic foundations and richer cultural and tourism resources consistently maintained higher levels of coordination throughout the study period. In contrast, provinces with weaker economic conditions or uneven internal development exhibited relatively lower coordination levels. Coordination levels evolved over time, and during 2018–2019 several provinces experienced notable improvements, significantly narrowing the gap between high- and low-coordination regions. This trend indicates a continuous strengthening of regional integration capacity.

From a stage-based perspective, improvements in coupling coordination followed a clear stepwise pattern. Between 2010 and 2011, coordination levels in most regions were relatively low, suggesting that inter-industry interaction was still in an early phase. During 2014–2015, coordination increased across all provinces, with several regions experiencing substantial improvements. By 2018–2019, most provinces had reached medium-to-high levels of coordination. This suggests that policy support, industrial upgrading, and cross-sector integration mechanisms had begun to generate sustained coordination effects.

Overall, Figure 5 demonstrates that coordinated development among the cultural, tourism, and sports industries did not emerge automatically from inter-industry linkages alone. Instead, it represents an evolutionary process characterized by cumulative effects and pronounced regional differences. The observed pattern—overall improvement coexisting with spatial heterogeneity—highlights the critical role of development foundations and structural alignment.

### 4.3 Driving factors and configuration mechanisms of coordinated development in cultural, Tourism, and sports industries

To further identify the drivers and internal mechanisms of coupling coordination, this study integrates an obstacle factor diagnostic model with fuzzy-set qualitative comparative analysis (fsQCA). This combined approach enables a systematic examination of the causal mechanisms and heterogeneous pathways through which regions achieve coordinated development.

#### 4.3.1 Analytical framework of influencing factors

Fundamentally, the integrated development of the cultural, tourism, and sports industries is shaped by multiple factors, including economic foundations, industrial structure, innovation capacity, and the institutional environment. Previous studies suggest that economic development determines regional demand conditions, while industrial structure strongly influences resource allocation efficiency. Technological innovation serves as an internal driver of industrial integration, whereas government governance plays a guiding role through policy coordination and institutional support. Drawing on theories of regional development, industrial upgrading, innovation-driven growth, and governance, this study constructs an integrated analytical framework. The framework comprises four dimensions: economic development level, industrial structure, technological innovation capacity, and government administrative capacity. This framework provides a coherent theoretical and empirical basis for subsequent configuration analysis.

Following principles of completeness, objectivity, and data availability, six indicators are selected as condition variables. Per capita GDP and per capita consumption expenditure capture regional economic development and purchasing power. The share of tertiary industry value added in GDP reflects industrial structure. R&D expenditure and the number of granted patents measure technological innovation capacity. General public budget expenditure serves as a proxy for government administrative capacity and policy support (Table 3).

**Table 3:** Indicator system of influencing factors for coordinated development of cultural, tourism, and sports industries in the middle and lower reaches of the Yellow River.

Influencing factor	Indicator/measurement	Variable	Implication/interpretation
Level of Economic Development	Per Capita GDP (CNY)	X1	Living Standards of Residents
Residents' Consumption	Per Capita Consumption Expenditure (CNY)	X2	Consumption Level of Residents
Industrial Structure	Proportion of Tertiary Industry in GDP (%)	X3	Level of Industrial Chain Integration
Scientific & Technological Capability	R&D Expenditure (10,000 CNY)	X4	R&D Input Capacity
Scientific & Technological Capability	Number of Granted Patents (item)	X5	S&T Output Capacity
Administrative Capacity	General Public Budget Expenditure (10,000 CNY)	X6	Government Investment Capacity

#### 4.3.2 Diagnosis of obstacle factors affecting coupling coordination: the case of Inner Mongolia autonomous region

Based on the previously calculated coupling coordination levels of the cultural, tourism, and sports industries across provinces in the middle and lower reaches of the Yellow River, overall coordination remains relatively low. To identify the key constraints on coordinated development, obstacle factor diagnostics were conducted for the period 2010–2019, and the five most influential constraints were identified for each stage (11)–(13).

As shown in Table 4, the obstacle factors constraining coordinated development in Inner Mongolia evolved substantially between 2010 and 2019. The dominant constraints shifted from limitations in tourism capacity and resource availability to insufficient factor supply and low levels of marketization. This transition indicates an evolution from scale-based constraints toward structural and efficiency-related constraints in regional industrial integration.

**Table 4:** Major obstacle factors affecting the coordinated development of cultural, tourism, and sports industries in Inner Mongolia.

Year/period	Primary obstacle factor	Secondary obstacle factor	Tertiary obstacle factor	Fourth obstacle factor	Fifth obstacle factor
2010—2011	C9 (8.27%)	C11 (7.66%)	C12 (7.58%)	C14 (6.65%)	C10 (6.38%)
2012—2013	C9 (9.00%)	C11 (7.74%)	C12 (7.51%)	C10 (6.59%)	C16 (6.35%)
2014—2015	C17 (9.29%)	C11 (7.81%)	C16 (7.73%)	C10 (7.53%)	C12 (6.69%)
2016—2017	C17 (10.21%)	C2 (8.79%)	C19 (8.24%)	C10 (7.91%)	C5 (7.89%)
2018—2019	C5 (13.05%)	C20 (11.80%)	C4 (10.80%)	C6 (10.69%)	C17 (10.17%)

##### 4.3.2.1 Early stage (2010–2013): constraints driven by tourism foundations and visitor scale

During 2010–2011 and 2012–2013, the number of A-level scenic sites (C9), domestic tourist arrivals (C11), and international tourist arrivals (C12) were the primary constraints on coordination among the cultural, tourism, and sports industries. This indicates that early-stage integration was mainly limited by insufficient tourism attractions and a relatively small tourism market. Although Inner Mongolia possesses distinctive grassland culture, ethnic heritage, and ecological resources, its remote location, limited transport accessibility, and homogenization of attractions have resulted in a shortage of high-quality and differentiated scenic sites. This weakens the formation of stable visitor flows. Prior studies show that in less developed regions, inadequate tourism infrastructure and core attractions significantly reduce tourism's spillover effects on related industries, thereby constraining early-stage industrial integration (37). In addition, the limited number of international visitors restricted external linkages between cultural presentation, sports events, and tourism consumption. As a result, inter-industry collaboration largely remained at the level of simple resource aggregation, making high value-added integration difficult to achieve.

##### 4.3.2.2 Middle stage (2014–2017): insufficient supply of cultural and sports inputs

During 2014–2015 and 2016–2017, the main constraints on coordination among the cultural, tourism, and sports industries in Inner Mongolia shifted from limitations in tourism foundations and visitor scale to shortages in cultural and sports inputs. In particular, the importance of the number of certified athletes (C17), sports social organizations (C16), cultural centers (C2), and employees in the sports system (C19) increased markedly. Despite strong policy support, the supply of sports professionals, social organizations, and grassroots public cultural facilities remained insufficient. Previous studies indicate that during the evolution of tourism- sports integration, shortages in sports human capital and organizational resources severely constrain event branding and linkages with the tourism sector (38). Similarly, inadequate public cultural facilities and social cultural organizations hinder deeper integration between the cultural industry and other sectors (39). This structural imbalance—abundant resources but weak organizational capacity—kept industrial integration at a low value-added stage. Over time, a pattern characterized by “rich resources, weak organizations, limited activities, and low conversion efficiency” became increasingly evident.

Although sports events and cultural activities expanded significantly, shortages of professional talent, social organizations, and grassroots public cultural facilities became more pronounced. On the one hand, the lack of high-level sports professionals constrained the branding of local sports events and their integration with tourism. On the other hand, the limited number of cultural activities and sports social organizations led to a growing mismatch between resource endowment and effective transformation.

##### 4.3.2.3 Late stage (2018–2019): market development and managerial capacity as key constraints

During 2018–2019, the main constraints shifted from internal shortages within the cultural and sports sectors to limitations in market development and managerial capacity. Key limiting factors included employment in the cultural sector (C5), operating revenue from sports activities (C20), the number of cultural market entities (C4), and operating profits in the cultural industry. The results indicate that market maturity and firm-level managerial capacity gradually replaced factor inputs as the main drivers of industrial performance and coordination quality (40). Although the cultural, tourism, and sports industries in Inner Mongolia expanded substantially in physical scale, they failed to generate stable market returns. First, the limited number and small scale of market participants constrained value-chain expansion. Second, weak profitability restricted reinvestment, causing inter-industry collaboration to rely heavily on government funding and undermining long-term sustainability.

Overall, from 2010 to 2019, the constraints on coordinated development among the cultural, tourism, and sports industries in Inner Mongolia followed a stepwise transition from “resources” to “inputs” and ultimately to “markets”. Early development was constrained by tourism resource utilization and visitor scale, the middle stage by shortages in cultural and sports inputs, and the later stage by weak market development and profitability. This trajectory closely aligns with Inner Mongolia's regional characteristics—rich resource endowments, geographic remoteness, and low population density—indicating that industrial coordination is not a process of simple expansion but a long-term transition shaped by multiple structural constraints.

#### 4.3.3 Configuration pathways of coupled and coordinated development among the cultural, Tourism, and sports industries — a fuzzy-set qualitative comparative analysis (fsQCA)

Using the obstacle factor diagnostic model, this study evaluates the level of coordinated development among the cultural, tourism, and sports industries in the middle and lower reaches of the Yellow River, systematically identifying the conditions that constrain inter-industry coordination. The results indicate that these constraints evolve dynamically, shifting from insufficient industrial output to low efficiency in factor allocation and inadequate institutional support. While this analysis clarifies which factors hinder coordinated development, it does not fully explain how different factors interact across regional contexts to produce high levels of coupling and coordination.

#### 4.3.4 Calibration of condition and outcome variables

All variables were first transformed into fuzzy-set membership scores ranging from 0 to 1. The direct calibration method was applied to the original continuous variables, with thresholds set at 0.95 for full membership, 0.55 as the crossover point, and 0.05 for full non-membership. Fuzzy-set membership scores were derived using a logistic calibration function following the direct calibration approach proposed by Ragin. The transformation from raw values to fuzzy-set membership scores is defined as:

$$S(x)=\frac{1}{1+\exp[-k(x-c)]}$$ (14)

where S(x)S(x)S(x) represents the membership score of case xxx in the corresponding set, xxx denotes the raw value of the variable, ccc is the crossover point, and kkk is a scaling parameter determined by the full membership and full non-membership thresholds. In this study, the calibration anchors were set at 0.95 (full membership), 0.55 (crossover), and 0.05 (full non-membership).

Based on a comprehensive consideration of theoretical logic and empirical distribution characteristics of the data, three qualitative anchor points were set, with crossover points set near the median of the variable samples, to represent the uncertain state of set membership and non-membership to the greatest extent. At the upper and lower tail ends of the distribution, threshold values for complete membership and complete non-membership were anchored. Due to the small sample size in this study, this calibration method based on distribution characteristics is particularly applicable. It not only maintains the differences between different provinces but also avoids the loss of information caused by arbitrary dichotomy.

The condition variables capture key dimensions of regional development, including economic development (GDP per capita and urban per capita consumption expenditure), industrial structure (the share of the tertiary sector in GDP), technological innovation capacity (R&D expenditure and number of granted patents), and government support intensity (general public budget expenditure). The outcome variable is the level of coupled and coordinated development among the cultural, tourism, and sports industries (Table 5).

**Table 5:** Fuzzy-set membership scores of variables.

Region	X1	X2	X3	X4	X5	X6	D
Inner Mongolia	0.71	0.85	0.05	0.03	0.04	0.04	0.03
Shanxi	0.03	0.04	0.05	0.19	0.06	0.08	0.25
Shaanxi	0.49	0.39	0.20	0.23	0.35	0.59	0.20
Henan	0.16	0.10	0.84	0.79	0.77	0.97	0.96
Shandong	0.97	0.96	0.97	0.97	0.97	0.48	0.92

X1 = per capita GDP; X2 = per capita consumption expenditure of Urban residents; X3 = proportion of the tertiary industry in GDP; X4 = R&D expenditure; X5 = number of granted patents; X6 = general public budget expenditure; D = coupling coordination degree of the culture, Tourism, and sports industries.

#### 4.3.5 Necessity analysis of single conditions

This section first examines whether any single condition constitutes a necessary prerequisite for achieving a high level of coupling coordination. According to established fsQCA standards, a condition is considered necessary only when its consistency exceeds 0.90.

On this basis, the study further assesses whether any individual condition is required for attaining a high level of coupling coordination. In line with international quality assessment criteria, only conditions with consistency values above 0.90 can be regarded as necessary. Following fsQCA guidelines, the necessity of individual conditions was tested. As shown in Table 6, none of the examined conditions achieved a consistency value exceeding 0.90. This finding indicates that no single economic, structural, technological, or institutional factor is indispensable for high-level coupling coordination.

**Table 6:** Necessary condition test for high coupling coordination degree among cultural industry, tourism industry, and sports industry.

Condition variable	High coupling coordination		Non-high coupling coordination	
	Consistency	Coverage	Consistency	Coverage
Per Capita GDP (X1)	0.567797	0.562431	0.511364	0.572034
∼Per Capita GDP	0.572034	0.511364	0.613636	0.613636
Per Capita Urban Consumption Expenditure (X2)	0.546610	0.551282	0.530303	0.598291
∼Per Capita Urban Consumption Expenditure	0.601695	0.533835	0.602273	0.597744
Proportion of Tertiary Industry in GDP (X3)	0.864407	0.866825	0.159091	0.199052
∼Proportion of Tertiary Industry in GDP	0.283898	0.231834	0.873485	0.889273
R&D Expenditure (X4)	0.802542	0.863801	0.215909	0.257919
∼R&D Expenditure	0.305085	0.258065	0.869697	0.817563
Number of Granted Patents (X5)	0.838983	0.804110	0.215909	0.260274
∼Number of Granted Patents	0.313559	0.263345	0.820455	0.864769
General Public Budget Expenditure (X6)	0.741525	0.810185	0.314394	0.384259
∼General Public Budget Expenditure	0.436441	0.362676	0.844697	0.785211

fsQCA, the “∼” symbol denotes the logical operation “NOT”, indicating the absence or low level of that element.

In line with established configurational analysis conventions, core conditions are those that appear in both the parsimonious and intermediate solutions, indicating their robustness under different logical assumptions. Contributing conditions, by contrast, do not appear in the parsimonious solution but are present in the intermediate solution. This distinction makes it possible to identify structurally necessary key conditions within the configuration.

### 4.4 Configuration pathways of coordinated development in the cultural, Tourism, and sports industries

Based on the preceding analysis, no single necessary condition for achieving a high level of coupling coordination was identified. Accordingly, this study employs configurational analysis to uncover multiple pathways leading to high coupling coordination. By systematically comparing the complex, parsimonious, and intermediate solutions, and defining core conditions as the overlap between the parsimonious and intermediate solutions, two representative high-level coordination pathways are identified.

The results indicate that regions can achieve high coupling coordination through different combinations of conditions, further confirming the existence of multiple pathways for coordinated development across the cultural, tourism, and sports industries (Table 7).

**Table 7:** Configurations leading to high coupling coordination.

Conditional Variable	Configuration 1	Configuration 2
Per Capita GDP	✕	⊗
Per Capita Urban Consumption Expenditure	✕	⊗
Proportion of Tertiary Industry in GDP	●	●
R&D Expenditure	●	●
Number of Patents Granted	●	●
General Public Budget Expenditure	●	✕
Raw Coverage	0.864407	0.902542
Unique Coverage	0.021864	0.550848
Consistency	0.966825	0.963801
Solution Coverage:	0.908333	
Solution Consistency:	0.923729	

● Core condition present (causal necessity).

⊗ Contributing condition present (causal sufficiency).

✕ Condition absent.

() "Don’t care”—condition may be either present or absent.

#### 4.4.1 Configuration pathway 1: structure–innovation–fiscal support–led pathway

This pathway is characterized by a high share of the tertiary sector in GDP, strong R&D investment intensity, a large number of granted patents, and sufficient general public budget expenditure as core conditions, while per capita GDP and urban residents' consumption levels are not required. The results indicate that even in regions with relatively limited economic development and consumption capacity, a high level of coupling coordination among the cultural, tourism, and sports industries can be achieved when a sound industrial structure is supported by sustained technological innovation and effective fiscal support (41). This pathway highlights the importance of supply-side capacity building and institutional intervention, demonstrating that industrial structure optimization combined with policy support can promote coordinated multi-industry development in economically less-developed regions.

#### 4.4.2 Pathway 2: demand–structure–innovation–driven configuration

This configuration is characterized by high per capita GDP, strong urban consumption capacity, a high share of the tertiary sector, substantial R&D investment, and a large number of granted patents as core conditions for achieving high coupling coordination. It indicates that in regions with a strong economic base and robust market demand, the interaction among consumption-driven demand, industrial upgrading, and technological innovation can effectively promote deep integration of the cultural, tourism, and sports industries, even in the absence of direct fiscal intervention. This pathway highlights the dominant role of market mechanisms and innovation dynamics in driving coordinated industrial development.

Overall, coordinated development among the cultural, tourism, and sports industries does not rely on a single optimal pathway; different configurations can produce comparable outcomes under different conditions. Accordingly, regions may select context-specific configuration pathways based on their economic conditions, industrial structure, and innovation capacity to achieve high-quality coordinated development.

## 5 Conclusions

This study focuses on the middle and lower reaches of the Yellow River and moves beyond the traditional practice of treating the “coupling coordination degree” as a sole outcome indicator. It examines whether the coordinated development of the cultural, tourism, and sports industries can be achieved through multiple pathways, based on the premise that high-level coordination does not depend on a single factor but emerges from the combined effects of multiple conditions. The results show that the cultural, tourism, and sports industries in the middle and lower Yellow River region exhibit a pattern of high coupling with gradual improvement in coordination, alongside substantial interprovincial heterogeneity. No single necessary condition exists for achieving a high level of coordination; instead, such outcomes are realized through different configuration pathways, such as structure–innovation–institutional support or demand–structure–innovation.

At the theoretical level, by combining coupling coordination measurement with configurational analysis, this study demonstrates that coordinated development among the cultural, tourism, and sports industries is characterized by nonlinear and multi-causal relationships. It advances the understanding that different combinations of conditions can lead to the same developmental outcome. From a practical perspective, the findings suggest that regions can adopt differentiated development strategies based on their economic conditions, industrial structures, and innovation capacity, thereby providing empirical support for more targeted policies on cultural, tourism, and sports industry integration.

Several limitations should be acknowledged. First, the analysis is confined to the middle and lower Yellow River region, and the generalizability of the findings requires further testing. Second, due to data constraints, certain institutional contexts and micro-level behavioral factors could not be incorporated. Third, the use of period-averaged data limits the ability to capture short-term shocks and dynamic adjustment processes. Future research could extend the analysis across broader spatial and temporal scales by incorporating longer time series and micro-level data, and by applying dynamic configurational approaches or multi-method triangulation to gain deeper insight into the evolutionary mechanisms of multi-industry integration.

Overall, this study provides a comprehensive configurational explanation of the diverse pathways through which cultural, tourism, and sports industries achieve coordinated development in the middle and lower Yellow River region, offering new theoretical perspectives and practical insights into multi-industry integration under regional high-quality development.

## Data Availability

The original contributions presented in the study are included in the article/Supplementary Material, further inquiries can be directed to the corresponding author.

## Appendix A

TABLE A1 Interpolated observations in the dataset.

**Table:**

Indicator	Code	Province	Year	Method
Number of Libraries	C1	Shanxi	2011	Linear interpolation
Proportion of Cultural Expenditure in Fiscal Budget	C3	Inner Mongolia	2012	Linear interpolation
Operating Profit of Cultural Industry	C6	Henan	2013	Linear interpolation
Number of Travel Agencies	C7	Shaanxi	2014	Linear interpolation
Number of International Tourists	C12	Shandong	2014	Linear interpolation
International Tourism Earnings	C14	Inner Mongolia	2015	Linear interpolation
Sports Fiscal Expenditure	C15	Shanxi	2016	Linear interpolation
Number of Classified Referees	C18	Henan	2017	Linear interpolation
Operating Income of Sports Institutions	C20	Shandong	2018	Linear interpolation

A total of 11 missing observations were supplemented using linear interpolation based on adjacent years. These account for approximately 1.05% of the total dataset (1050 observations), which is well below the 5% threshold used in this study.
